# Diagnosis and management of hidradenitis suppurativa: Analysis of US insurance claims data

**DOI:** 10.1016/j.jdin.2023.10.002

**Published:** 2023-10-21

**Authors:** Betty Xiong, James Zou, Waqar Ali, Roxana Daneshjou, Jonathan Williams

**Affiliations:** a Department of Biomedical Data Science, Stanford University, Stanford, California; b Department of Biomedical Data Science and Dermatology, Stanford University, Stanford, California; c UCB Pharma, Slough, UK; d UCB Pharma, Brussels, Belgium

**Keywords:** claims analysis, delayed diagnosis, diagnosis, evidence-based medicine, hidradenitis suppurativa, medical specialty

*To the Editor:* Patients with hidradenitis suppurativa (HS) wait an average of almost 10 years to get a formal diagnosis, leading to significant pain, distress, and scarring.^1^^,^^2^ To better understand the patient journey from the initial diagnosis of HS, we utilized the PatientSource data set (July 1, 2008-January 31, 2022) from Symphony Health, an ICON plc Company. This is an interconnected source of prescription, medical, and hospital claims across payers, pharmacies, hospitals, and clinics. A 2% random sample from the PatientSource database, equating to 5.9 million individuals, was analyzed to identify patients with HS.

We define HS patients as having 1 or more claims of HS diagnoses^3^ using the International Classification of Diseases, Ninth Revision code L73.2 or 1 or more instance(s) of the 10th Revision code 705.83 between July 2010 and January 31, 2022. The index date is the date of the first identified diagnosis of HS. To capture disease history, it is required that patients have at least 2 years of claims data coverage prior to the index date, which is defined as at least 1 claim of any type (prescription, medical, or hospital) in each of the 2 years prior to the index date.

The analysis was performed on a final cohort of 8929 patients, with all 50 US states represented. The proportion of patients with HS in the data set was 0.3%, in line with statistics identified from published literature.^2^

To understand the patient journey, we assessed the physician specialty seen at first diagnosis, and second and third occurrence of HS diagnosis codes. Approximately 75% of the HS diagnoses in the data set have a physician specialty recorded. The average age at first diagnosis is 37 years (SD: 15.5 years), with male and female patients diagnosed at an average age of 42 and 36, respectively. Family medicine/general practice specialties make the highest percentage of first diagnosis (24%) followed by dermatology (21%). Other groups involved in making the first diagnosis include internal medicine, general surgery, and emergency medicine (Fig 1). However, after the first diagnosis, dermatologists become the leading specialist seen at the second occurrence (24% dermatology and 20% family medicine/general practice) and at the third occurrence (25% dermatology and 16% family medicine/general practice). After initial diagnosis and subsequent increase in care by dermatologists, the presence of emergency medicine in the top 5 specialties decreases (Fig 1), which could result in lower medical costs.

**Fig 1:**
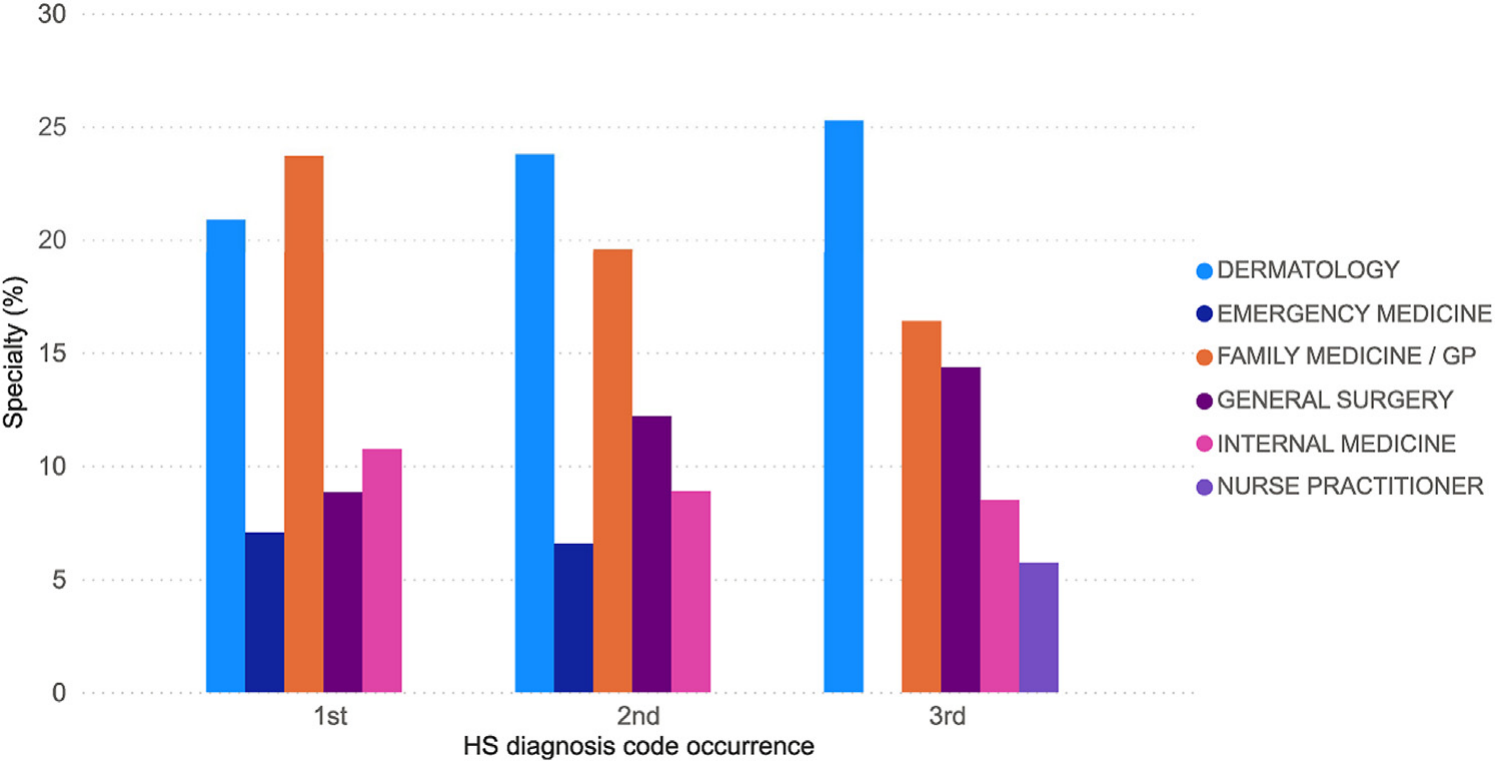
The chart visualizes the top 5 ranked specialties seeing patients at first diagnosis, and second and third occurrence of a hidradenitis suppurativa diagnosis code. At third occurrence of a hidradenitis suppurativa diagnosis code, nurse practitioner replaces emergency medicine as the fifth ranked specialty. *GP*, General practice; *HS*, hidradenitis suppurativa.

Given the results from this study, it is likely that patients have been seen many times by nondermatology specialists prior to diagnosis, therefore efforts should focus on improving diagnostic ability of these groups to increase rates and speed of referral to dermatologists.

Limitations of this study include potentially missing claims data (for determining initial diagnosis), and lack of granular data on disease severity at diagnosis. Future research should look at the correlation between severity of disease and specialty at first diagnosis.

## Conflicts of interest

Authors Ali and Williams are employees of UCB Pharma. Author Xiong, Dr Zou, and Dr Daneshjou have no conflicts of interest to declare.
